# Evaluating Change of Attendance Rates in Psychiatric Outpatient Clinics Following Introduction of Short Message Service in a Mental Health Service in West Midlands, England

**DOI:** 10.1192/bjo.2025.10507

**Published:** 2025-06-20

**Authors:** Mohamed Yaasir Mohamudbucus, Nilamadhad Kar

**Affiliations:** Black Country Healthcare NHS Foundation Trust, Wolverhampton, United Kingdom

## Abstract

**Aims:** It was intended to explore the change in nonattendance rate at outpatient clinics following the introduction of Short Message Service (SMS) reminders in the Black Country Healthcare NHS Foundation Trust, which serves four regions in the West Midlands.

**Methods:** The Trust introduced an SMS system in March 2024 to prompt the patients about their upcoming outpatient appointments. In a mirror-image design, we analysed the Did Not Attend (DNA) rates for 6 months pre and post-SMS introduction, from September 2023 to February 2024 and April 2024 to September 2024 respectively. All the patients offered an outpatient appointment were included in the data collection; with no exclusion. The study was approved by the Research and Innovation team of the Trust as a service evaluation.

**Results:** A total of 14094 appointments were taken into consideration before the introduction of SMS reminders, and a total of 14852 appointments were analysed post-SMS introduction. Before the introduction of SMS reminders, the average DNA rate across all four regions of the Trust was 22.8% (95% CI: 22.2–23.5) with a range of 19.9–24.8 in the six months. After the introduction of SMS reminders, the average DNA rate changed to 23.2% (95% CI: 22.5–23.8) with a range of 21.3–25.1 in the six months; and this change was statistically non-significant (NS). Two regions had an increase of DNA (21.1% to 21.9%, NS; and 20.7% to 24.7%, p<0.05) and others had a decrease (25.2% to 23.1%, p<0.05; and 24.7% to 23.6%, NS).

**Conclusion:** It appeared that within six months of the introduction of the SMS reminder system, there was no significant change in the DNA rates in the Trust; although there were regional variations of both increase and decrease in rates. There are multiple factors that can influence attendance at outpatient clinics such as accessibility, patient-related factors, and the effectiveness of a reminder system. It is also probable that the first six months may be early for the SMS system to establish its potential, and longer-term observational data might be needed. Similarly, the difference in DNA rates between regions cannot be explained without more in-depth data. There was a limitation in finding out whether all the patients were sent or received the reminders. There may be a scope to decrease the number of missed appointments through SMS, but further studies are required. In addition, the effectiveness of local processes of inviting patients and sending reminders needs to be checked.

